# DIPSBC - data integration platform for systems biology collaborations

**DOI:** 10.1186/1471-2105-13-85

**Published:** 2012-05-08

**Authors:** Felix Dreher, Thomas Kreitler, Christopher Hardt, Atanas Kamburov, Reha Yildirimman, Karl Schellander, Hans Lehrach, Bodo MH Lange, Ralf Herwig

**Affiliations:** 1 Department of Vertebrate Genomics, Max Planck Institute for Molecular Genetics, Ihnestr. 63-73, 14195, Berlin, Germany; 2 Institute of Animal Science, University of Bonn, Endenicher Allee 15, 53115, Bonn, Germany

**Keywords:** Data integration, XML, Data visualization

## Abstract

**Background:**

Modern biomedical research is often organized in collaborations involving labs worldwide. In particular in systems biology, complex molecular systems are analyzed that require the generation and interpretation of heterogeneous data for their explanation, for example ranging from gene expression studies and mass spectrometry measurements to experimental techniques for detecting molecular interactions and functional assays. XML has become the most prominent format for representing and exchanging these data. However, besides the development of standards there is still a fundamental lack of data integration systems that are able to utilize these exchange formats, organize the data in an integrative way and link it with applications for data interpretation and analysis.

**Results:**

We have developed DIPSBC, an interactive data integration platform supporting collaborative research projects, based on Foswiki, Solr/Lucene, and specific helper applications. We describe the main features of the implementation and highlight the performance of the system with several use cases. All components of the system are platform independent and open-source developments and thus can be easily adopted by researchers. An exemplary installation of the platform which also provides several helper applications and detailed instructions for system usage and setup is available at http://dipsbc.molgen.mpg.de.

**Conclusions:**

DIPSBC is a data integration platform for medium-scale collaboration projects that has been tested already within several research collaborations. Because of its modular design and the incorporation of XML data formats it is highly flexible and easy to use.

## Background

Systems biological research is frequently carried out within collaborations connecting multiple labs each conducting a specific type of experimental work. The ultimate goal of these research collaborations is the integrated analysis of the data generated within the consortium. Data integration involves the storage and cross-linking of initially independent and heterogeneous data sets. This allows for the simultaneous analysis of data sets and therefore enhances the overall functional interpretation, which provides additional information compared to the sequential analysis of single data sets [1-4]. An important prerequisite for data integration is the standardization of storage and exchange formats, both within data domains (e.g. mass spectrometers of different manufacturers) and across different data domains (e.g. mass spectrometry and DNA microarrays), since such data typically show a lack of coherence [5,6].

In this article we describe a data integration platform that provides a flexible representation of collaborative data based on XML. It is designed for research collaborations, typically involving heterogeneous 'omics' data along with functional data from validation experiments, genetic and phenotypic data. The introduction of new data types or the modification of existing data types can be easily accomplished, thus providing high format extensibility. This data representation approach takes advantage of a growing number of XML data formats in biotechnology [7-15].

The system is built upon three components: a) the web-server (Foswiki), providing a convenient user interface; b) the search index (Solr/Lucene), which can be accessed through the user interface, providing a fast full-text search engine; and c) helper applications (Java applets), providing interactive, data specific analysis functionality.

All components of the system are platform-independent, open-source developments, and thus can be easily adopted by researchers. An example installation of the collaboration platform with proto-typical public data sets is provided at http://dipsbc.molgen.mpg.de.

## Implementation

### General functionality

The functionality of the data integration system is realized by a combination of four components: XML, Solr/Lucene, Foswiki, and Java applets. In the following we describe the implementation and interplay of these components.

### Integration of existing and user-defined XML formats

**Figure 1:**
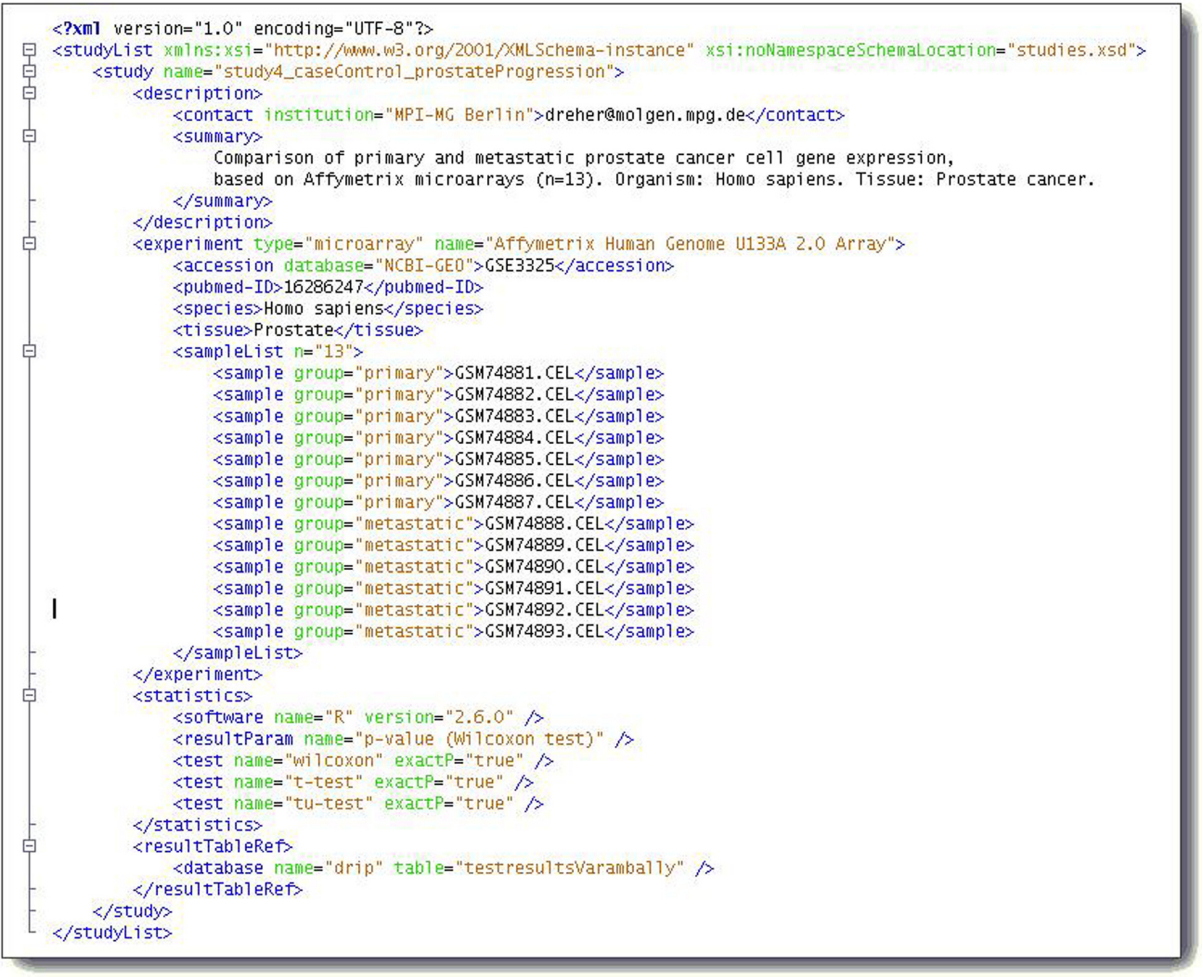
**XML code for a custom data type: 'study'.** This custom XML format represents data analysis results from transcriptome profiling based on DNA microarrays.

In recent years, several initiatives have specified and developed XML-based representations of primary data in domains such as proteomics, genomics, molecular interactions, cellular assays and mathematical models, amongst others [16-18]. XML features high format extensibility and can be used to represent virtually any kind of data structure, thus making it easy to integrate new data types and, importantly, modify existing ones. In addition, many tools in bioinformatics and systems biology use XML as their default exchange format. Besides the formats for primary data, XML offers the possibility of defining a structured representation of data analysis results. These results are typically adapted to a particular data analysis workflow and are highly user-specific, thus requiring a flexible data integration solution. An example for such a format is the XML schema that we defined for study results (Figure 1). Because of the advantages given above, DIPSBC uses XML for data representation, and a list of supported formats is given in Table 1.

**Table 1:** Standards initiatives and XML formats for different experimental technologies

**Data domain**	**Guidelines**	**Exchange format**	**Standards initiative**
Microarrays	MIAME	MAGE-ML, MINiML	MGED society (http://www.mged.org)
Mass spectrometry	MIAPE	mzML, mzData, mzXML	HUPO PSI-MS (http://www.psidev.info)
Molecular interactions	MIMIx	PSI-MI	HUPO PSI-MI (http://www.psidev.info)
In situ hybridization / Immunohistochemistry	MISFISHIE	MISFISHIE.dtd	MGED society (http://www.mged.org)
Cellular assays	MIACA	CAOM	MIACA Standards Initiative (http://miaca.sf.net)
Quantitative PCR	MIqPCR	RDM	RDML consortium (http://www.rdml.org)
Genomic sequences	MIGS	RDM	Genomics standards consortium (http://gensc.org)
Systems biology / Pathways	MIRIAM	SBML, CellML, BioPAX	Biomodels.net (http://biomodels.net/miriam)

The example installation of DIPSBC incorporates many of these data and allows integrated indexing of primary and secondary data.

### Data normalization and indexing

In a first data integration step in DIPSBC, all experimental data are converted to XML. The conversion can be done either by publicly available or by custom parsers. We use XML Schema Definition (XSD) in order to syntactically define the structure of the XML files and to ensure their data integrity. If available, community compliant XSDs like mzData (for mass spectrometry; [19]), MAGE-ML (for DNA microarrays; [20]), or PSI-MI (for molecular interactions; [21]) are used. This is an ultimate benefit since it ensures wide acceptance and compatibility of the data formats. For more specific data sets that lack community standards, custom schemas can be easily developed.

**Figure 2:**
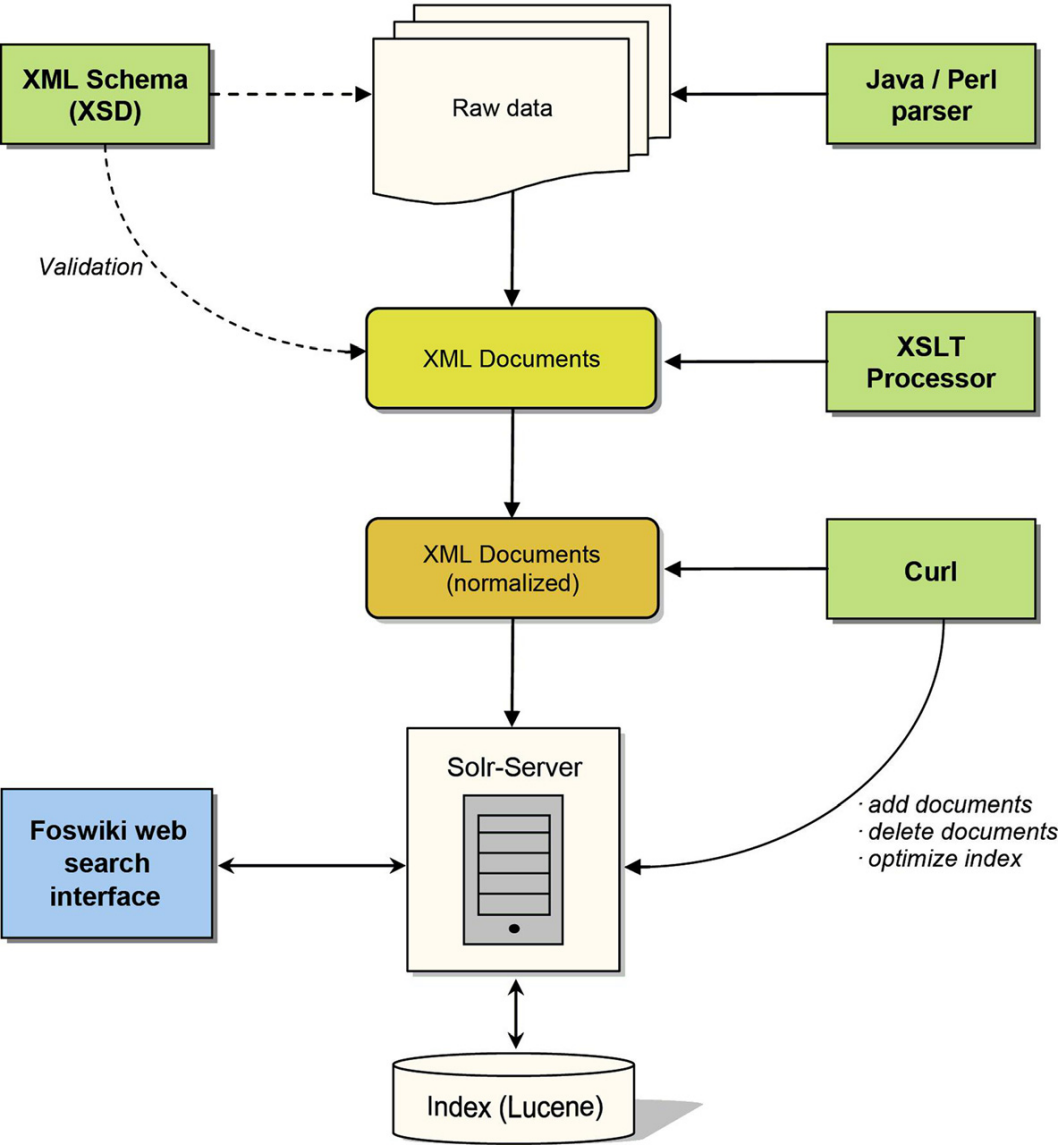
**Graphical representation of the data processing workflow**. Raw data are transformed to normalized XML files and indexed. The transformation is accomplished with Java or Perl parsers and XSLT. The integrity of XML files is ensured by XSD files. Normalized data sets are indexed with Solr/Lucene and can be queried via the web interface. 'Curl': command-line tool for the transfer of data from or to a server.

In order to add the data to the index and make them available for searching, all XML files are normalized, i.e. generalized versions of the files are created. The normalized files contain only the search-relevant content, stored in unified variables ('title', 'identifier', 'content', and 'annotation'). The normalization is done with XSLT (eXtensible Stylesheet Language Transformations), and the output can be directly fed into the Solr search index (Figure 2).

The search index can be created, updated, optimized, or queried through HTTP calls over the network. To increase the overall performance, the index server and the Foswiki web server can be installed on different computers. Additionally, Solr offers advanced query syntax and fast search routines. Usually response times remain below one second when querying the DIPSBC index, which contains about 35 million records (Table 2). Moreover, Solr/Lucene supports detailed configuration capabilities including custom document scoring functionality. As an example, we make use of this functionality when indexing gene expression results by using the fold-change and p-value of each gene as index score boost coefficients. Thus, the more differentially expressed a gene was in a study, the higher rank it will reach in the index result page.

**Table 2:** Index contents of the current DIPSBC example installation

**Data type**	**Source**	**File format**	**Nr. of entries**	**Description**
Protein mass spectra	PRIDE acc. 8538	mzData	745	Peptide tandem mass spectra (Homo sapiens) with identifications
DNA microarrays	GEO acc. GSE3325	MINiML	19	Prostate cancer study; chip platform: Affymetrix U133 Plus 2.0 arrays (Homo sapiens)
	GEO acc. GSE1133	MINiML	438	Novartis gene atlas 2004 (mouse and human arrays)
	GEO acc. GSE10204, GSE11193	MINiML	80	Genetic functional basics of water-binding- capacity in pork; chip platform: Affymetrix Porcine Whole Genome Array
Studies	MPI Berlin	XML 'study'	7	Summary tables of statistical analyses
Test result tables	MPI Berlin	STAT-ML	94497	Results of statistical analyses of microarray experiments
Microsatellite markers / phenotypes	University Bonn	XML 'pigs'	873	Pig marker and trait values
Molecular interactions	IntAct	PSI-MI	5915	Yeast-2-hybrid datasets from Rual et al. and Stelzl et al.
	CPDB	XML 'cpdb'	46454	Interactions involving genes, proteins, and compounds; source: ConsensusPathDB
Molecular Models	BioModels	SBML	699	Mathematical models of gene regulatory pathways
Synonyms pig	Affymetrix	XML 'synonyms'	24123	Pig genome annotations
Synonyms human	Affymetrix	XML 'synonyms'	54675	Homo sapiens genome annotations
Protein sequences	Uniprot	FASTA	16.5 mio.	Protein sequences (FASTA format)
Publications	PubMed	XML 'pubmed'	18.2 mio.	Publications in PubMed starting from 1970
Foswiki pages	DIPSBC	TXT	26	Web pages within the DIPBSC platform
Total nr. of entries			34.970.538	

The index contains a large collection of different data types, including protein mass spectra, DNA microarray experiments, molecular interactions, protein sequences and Pubmed abstracts, amongst others. In total about 35 million records are indexed and thus searchable.

**Figure 3:**
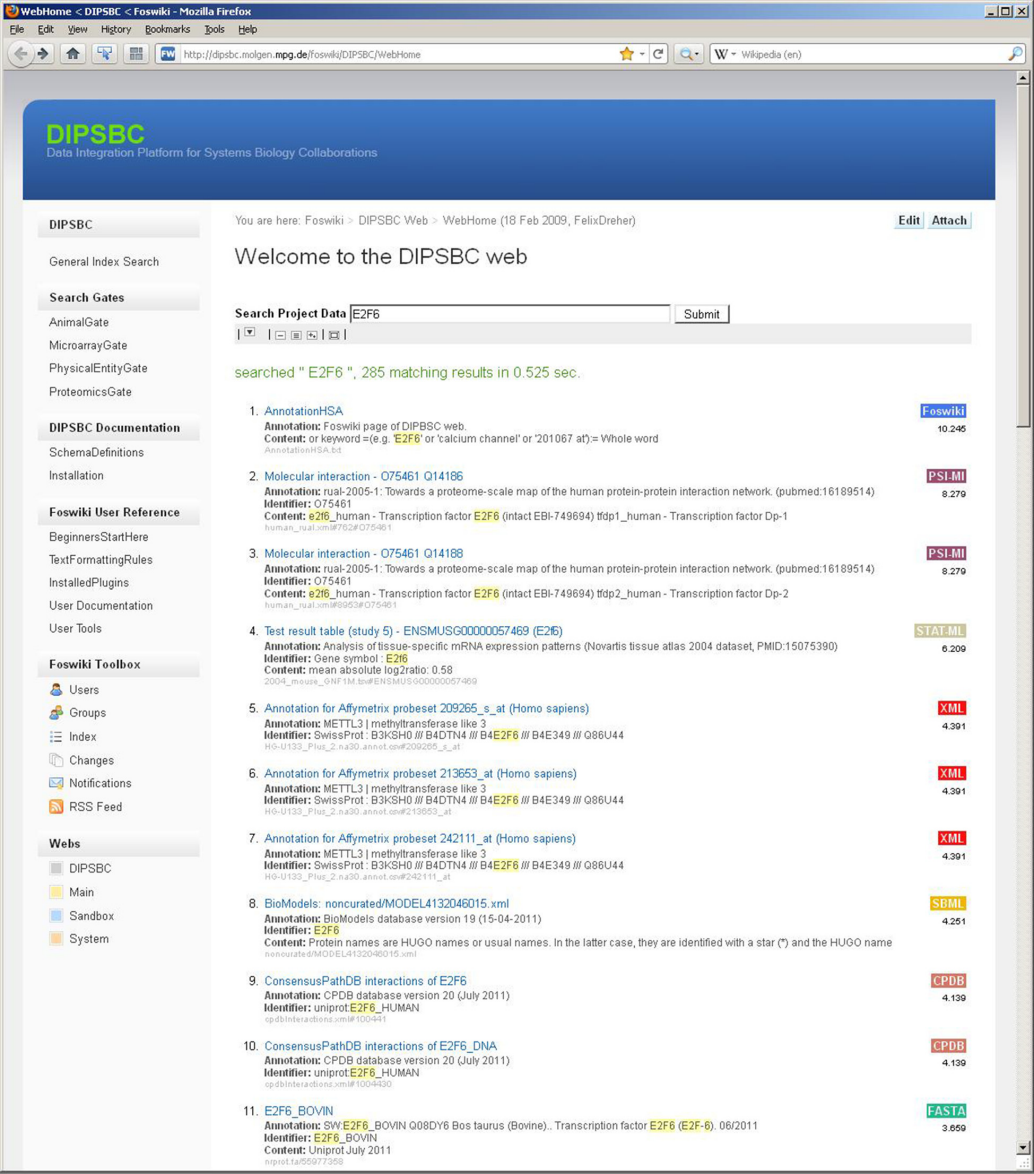
**DIPSBC search interface.** The result list for the exemplary query keyword "E2F6" is shown. Different result data types are indicated by colored icons and are linked to respective helper applications.

Besides possible boost values, the search result page orders the documents by contextual relevance. The sources file types (e.g. text, pdf, image, HTML, XML etc.) and additional links to further information are shown (Figure 3). Depending on the file type, either a report page or specific helper applications can be opened allowing a closer inspection and analysis of the data set. Consequently, all data sets can be searched simultaneously and be evaluated in context.

### Foswiki collaboration platform and incorporation of helper applications

DIPSBC uses the Foswiki content management software as a browser-independent user interface due to its advanced features for managing collaborations. For example, users can create or edit web-pages within their browser and directly upload and share data. All modifications made to the website or attached files are tracked by a built-in revision control system. Therefore, different document versions can be compared; moreover, e-mail notifications that automatically inform users about document changes can be enabled. Additionally, the Foswiki technology provides a fine-grained user management system, which can be used to define rights for viewing or editing web pages for different users or user groups. For more general data privacy, password-protection or IP-range checks at the web server level can be applied as well.

An important feature of the proposed platform is the possibility of a straightforward incorporation of helper applications associated with the different data types. For this purpose we take advantage of the Foswiki plug-in interface to integrate specialized programs as Java applets, resulting in minimal installation efforts on the client side, as the applets are automatically started within the user's web browser. Currently the system includes three different applets: the Argo Genome Browser [22] and two custom developed applets: an mzData viewer, which provides a graphical representation of peptide spectra, and a graph browser, which reads molecular interaction data stored in PSI-MI files and dynamically visualizes the underlying protein-protein interaction networks (Results and Discussion).

## Results

Here, we illustrate the usage of the system with several archetypical use cases that incorporate different levels of integrated primary data.

### Integration of experimental results from proteomic and transcriptomic data

Nowadays large-scale profiling on mRNA and proteome levels has become routine and increasing numbers of large-scale data sets have become available. A combination of these different experimental approaches will help to gain a more comprehensive view of biological processes and molecular networks [23-25]. Observing evidence of genes (proteins) in different heterogeneous data sets might lead to better disease markers. Data integration systems give a first glance in searching through these data sets. As an example, we used our data integration system to screen a prostate cancer gene expression study together with a mass spectrometry study of the Human Plasma Proteome Project II. In general, plasma proteome data sets could be used to identify biomarkers for certain disease states, as proteins up-regulated in diseased tissues may enter the blood stream in higher concentrations than usually [26]. Outside of the platform, we analyzed mRNA expression differences between primary and metastatic prostate cancer cells of the transcriptomic study [27], GEO accession GSE3325 statistically with R and identified 5,142 differentially expressed genes (Wilcoxon-test, P < 0.01; GCRMA normalization).

We then added these study results to the Solr index. An overview of the results, including download links to the test result tables, can be found by entering 'vindex:studies study 4' in the index search field. Likewise, genes differentially expressed in the study can most easily be found by entering 'study 4' in the index search field. Because the genes' search score is boosted according to the respective log2ratio and p-value, the most significant genes will be listed on top of the result page.

**Figure 4:**
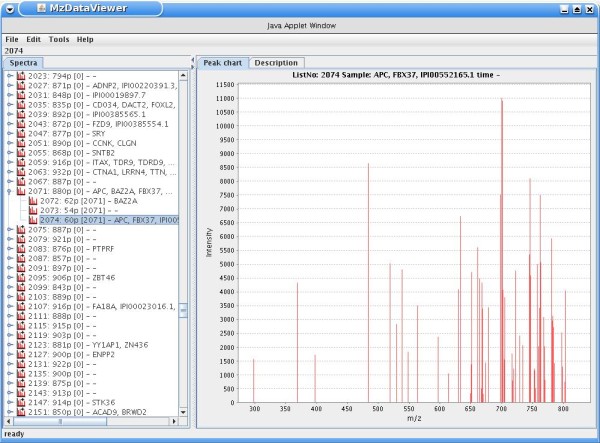
**Screenshot of the 'mzData viewer' applet.** This helper application can be used to visually examine results of peptide mass spectrometry experiments.

Several of the differentially expressed genes are known to play a critical role in prostate cancer, such as *APC*[28], *MAPK7*[29], or *ZEB1*[30]. Therefore, we correlated these genes with their identification in the human plasma proteome by querying the index for significant genes and checking the result list for mass spectrometry hits. In the case of the above mentioned three examples, these are found in the blood plasma sample as well. Clicking on the result link of a mass spectrum opens the 'mzData Viewer' applet, which can be used to view and zoom into the spectrum and display associated annotation (Figure 4). Additionally, protein identifications can be re-analyzed by uploading the respective XML file containing the spectrum's peak list to the public Mascot [31] web search interface.

### Characterization of candidate genes for an animal genome with sparsely known functional information

**Figure 5:**
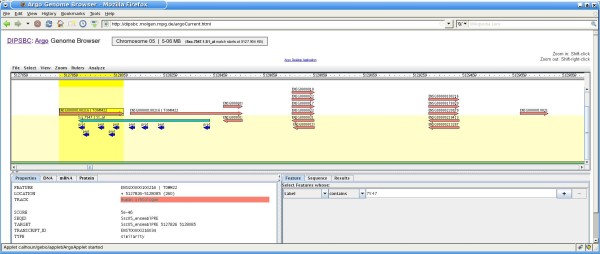
**Screenshot of the 'Argo Genome Browser' applet.** This helper application [22] provides a graphical representation of genomic regions with the respective features and annotations.

Many research projects in functional genomics are focusing on organisms whose genomes are still partly unknown. As a use case we investigated how functional information for porcine genes can be extrapolated with the architecture. The pig genome was only partly sequenced until recently, which made it difficult to identify genes which influence specific traits and the same holds still true for many other animal genomes. Therefore homology is a valuable concept which can help to find out more about possible functions of yet poorly characterized pig genes [32]. For example, based on an F2 resource population consisting of 873 animals, a QTL for the phenotypic trait ‘*drip loss*’ was identified on chromosome 5 [33] with the respective orthologous region in human located on chromosome 22 [34]. By alignment of porcine Affymetrix probes to the human genome we identified 137 pig genes that match to this region [35]. We carried out a gene expression analysis with animals of different genotypes with respect to this QTL and stored the statistical results in the DIPSBC index. As an example, we explored the role of one of these genes (UniGene-ID 'Ssc.7547'), which was found to vary significantly across different genotypes (ANOVA P-value < 0.03). Because the respective genomic region had been sequenced already, the 'Argo Genome Browser' applet could be used to inspect this region indicating that the gene lies within the mentioned QTL on porcine chromosome 5 (first, we searched the index for 'Ssc.7547', then clicked on the first hit and then on 'Genome Browser view'). Most importantly, human exons that match the pig gene have also been incorporated in the system. These exons belong to the human gene *TOMM22* which is a central receptor component of the mitochondrial translocase (Figure 5). To reveal molecular interactions of this gene, we searched the index and used the link to the ConsensusPathDB [36]. The resulting network shows a highly conserved interaction of *TOMM22* with *VDAC1* (voltage-dependent anion-selective channel protein 1). This protein forms a channel through the mitochondrial and plasma membrane, respectively, and is involved in molecule diffusion and cell volume regulation. Therefore, it probably also influences the specific trait under analysis and provides an interesting candidate for further validation.

### Querying protein-protein interactions based on two different network datasets

**Figure 6:**
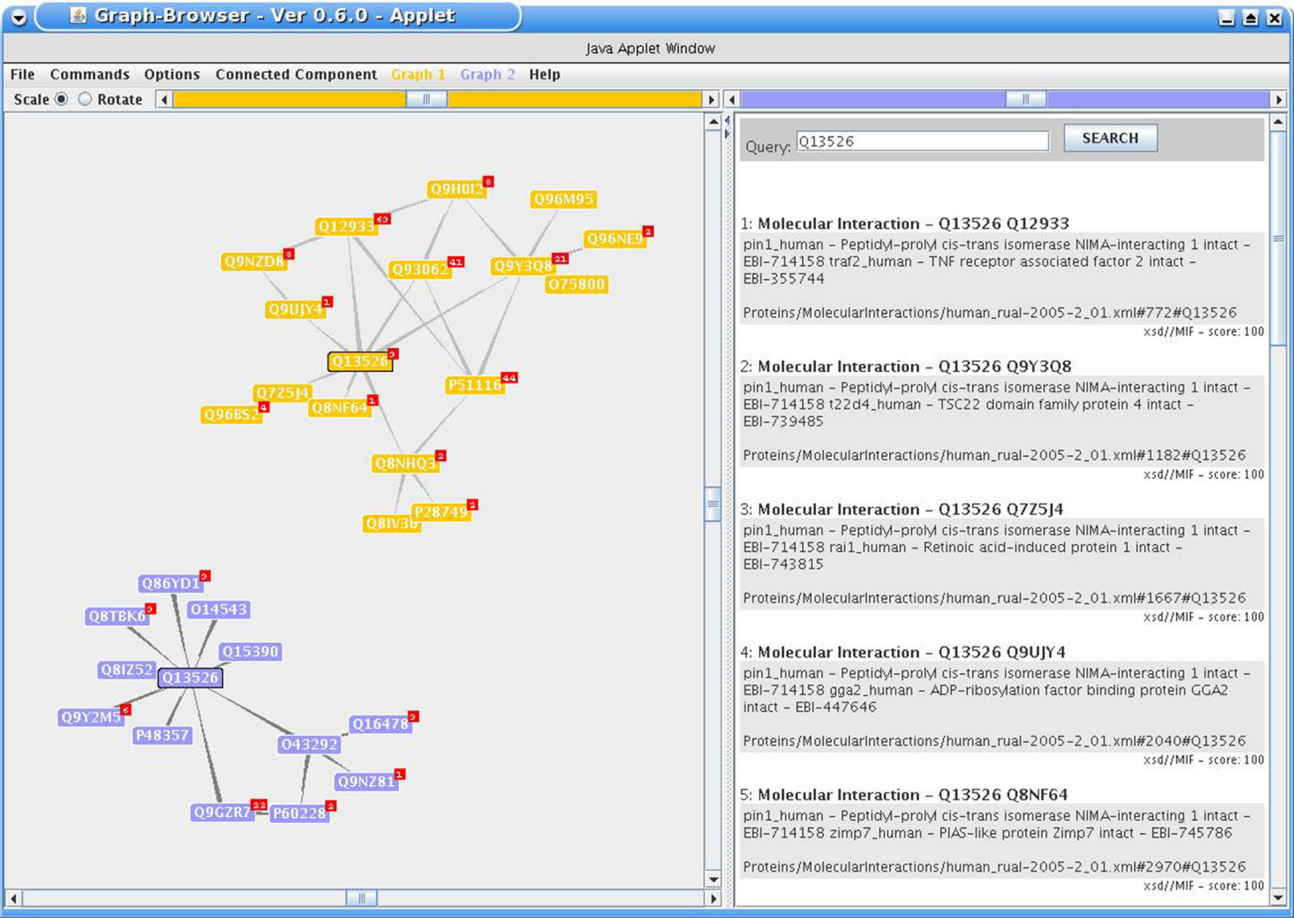
**Screenshot of the 'Graph Browser' applet.** This helper application can be used to visualize protein-protein interaction networks. Nodes represent proteins, edges represent interactions. Nodes can be expanded and collapsed, and meta-data can be accessed by clicking on individual nodes.

We integrated two datasets of human protein-protein interactions (PPIs), both constituting a representative part of the human interactome [37,38]. In each case, the authors performed systematic yeast two-hybrid (Y2H) screens that resulted in 2,671 and 3,129 interactions, respectively. The datasets are publicly available and we downloaded them as PSI-MI XML files from the IntAct database [39]. After normalization and indexing, an index query for a protein of interest lists all its interactions existent in the two datasets. By clicking on one of the interactions, the 'Graph browser' applet is launched, which parses the underlying original XML file and visualizes a sub-network around the focus protein. In case the queried protein is available in both interaction datasets, two sub-networks are generated side-by-side. This allows users to compare the two network topologies and to detect differences or overlaps. As an example, the gene *PIN1*, which is known to play a critical role in prostate cancer [40], has no overlapping direct neighbors in the two networks (Figure 6). Therefore the two graphs complement each other and by the analysis of both networks in parallel, additional interactions can be found. Furthermore, network visualizations can be inspected in greater detail and modified dynamically. Nodes and edges can be moved, added, and hidden. Additionally, each node can be expanded or collapsed, provided it is connected to other nodes; the radius of nodes shown in relation to the central protein can be defined as well. By clicking on a node, the Solr index is queried for the respective protein and the results are shown at the right of the application window.

### Linking genes to interaction networks and computational models

**Figure 7:**
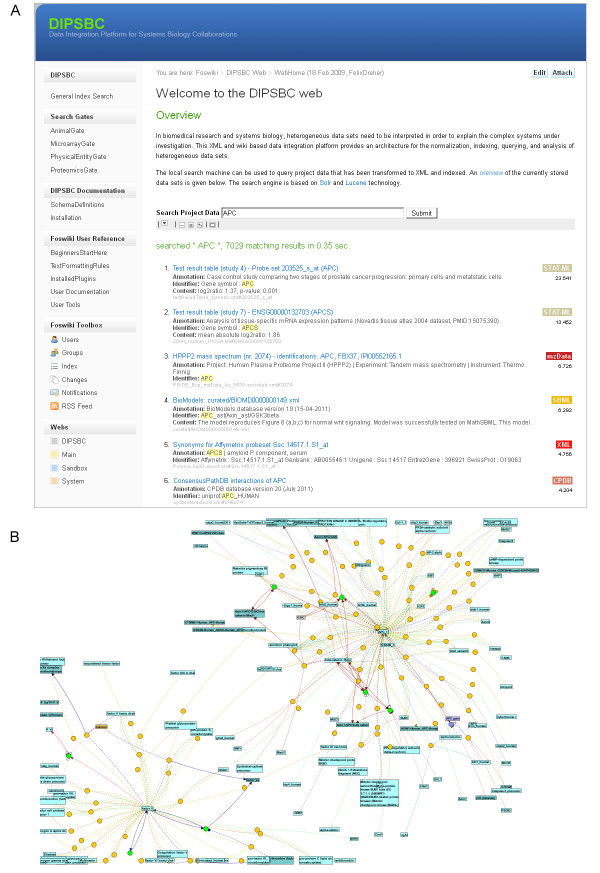
**Result page and network visualization for the APC gene.** A) Top search results for an exemplary query for the APC gene. Different experiment types are shown, ordered by relevance. B) Visualization of the interaction network around the APC gene.

Systems Biology studies specifically aim at interpreting biological data at the network level. Thus, a data integration system should be able to cross-reference primary data with interaction resources and computational models. This is demonstrated with the *APC* gene found significantly differentially expressed in the prostate cancer study mentioned in the first use case [27]. The index search results for the *APC* gene are displayed in Figure 7A; these results cover primary data records such as mass spectra, data analysis results from microarray studies, sequencing results as well as information on associated networks. As far as the latter are concerned, we indexed the BioModels database [41] and the ConsensusPathDB [36]. Indexing has been done with SBML documents in the former and with a specific XML format in the latter case (ConsensusPathDB). The first SBML hit of the APC index search directs the user to a computational model of Wnt/ERK signalling [42] stored in BioModels. The respective ConsensusPathDB entry links the user to the list of known molecular interactions of *APC* recorded in the database. Using the visualization function of ConsensusPathDB the interaction neighborhood of *APC* is displayed (Figure 7B). Thus, the architecture can be used to directly link primary data with biological networks.

### Comparison to related data integration systems

There are several related software tools for the integration of heterogeneous genome data, such as the ISA Infrastructure [43] and the BioMart system [44].

ISA Infrastructure consists of several Java desktop applications and a relational database, built around the ISA-Tab format. Amongst others, the system provides tools for metadata structure definition (ISAconfigurator) and data input and processing by collaborating experimentalists (ISAcreator). Experiment metadata is stored in the generic ISA-Tab format and can be exported to XML-based, community compliant formats to meet the standards of public repositories like ArrayExpress, PRIDE or European Nucleotide Archive (ENA). The system is well suited for the production of standardized, richly annotated experimental data and its formal validation. However, in comparison to DIPSBC, the system's data analysis and visualization options are rather small yet. Also, it has a less strong focus on the collaboration platform as has been realized in DIPSBC by incorporating the Foswiki system and its features.

BioMart is a data management system aiming at the integration of disparate, geographically distributed data sources. Typically the latter are relational databases, each maintained independently and with its own data structure. BioMart provides a consistent graphical user interface for the unified query of all contained sources. These can be filtered by different attributes, e.g. genomic region or gene ontology term. BioMart is used by several large-scale research consortia, e.g. the International Cancer Genome Consortium (ICGC) [45]. In general, the system is best suited for readily processed, i.e. finished data and its decentralized structure leads to a lightweight installation. However, it is less well suited to integrate complex, evolving data types that change frequently as is the case in particular at the start of new collaborative projects. Furthermore, it neither features a document sharing option nor the possibility of an index based full-text search.

Overall, both ISA infrastructure and BioMart are systems that are well suited for rather large collaboration projects, at the cost of increased time consumption and man-power. In comparison, our system has the advantage of being very flexible and extensible. In contrast to systems based on relational databases, our XML index based platform offers a straightforward way of integrating data sets via common keywords, supported by a very fast full-text search.

## Discussion

We presented a data integration system that is utilizing and indexing XML-based data representation formats. Thus, the basic unit of data stored within the DIPSBC platform is 'XML document'. This unit is very generic and can range from genes and pathways to whole genome microarray experiment results, implicating a very high variability in data granularity. We use XML as central data format in order to capture this granularity and to make heterogeneous data compatible, a prerequisite for the coordinated integration of the various data sets.

As a result, the document management of our system is highly flexible, community compliant and well suited for data collaborations. On the one hand, the adoption of community standards enables cross-referencing proprietary data with publicly available data sets and applets for data visualization such as genome browser etc. as was demonstrated in the use cases. On the other hand, in particular with data types that are not yet standardized or that are so heterogeneous that they can not be standardized, for example the very specific data analysis results, the system offers full format flexibility and has basically no restrictions as was demonstrated by introducing a custom standard for data analysis results (Figure 1).

Currently the procedure of adding new data to the system involves two steps: first, the member of the consortium who generated the data set (e.g. from a microarray experiment) transfers it to the administrator. Second, the administrator checks the data for integrity by XSD schema validation and then adds the normalized XML to the index. Although this procedure ensures improved data integrity by manual curation, it would still be favourable to automate the procedure of XML transformation, validation, normalization and indexing, for example by implementing custom Perl plugins. These plugins could provide data upload interfaces, enabling members of a given collaboration to directly add their experimental data to the system. A corresponding interface is currently under development and will be provided in a future version of DIPSBC.

In the age of 'omics'-data, researchers are faced with ever growing data set sizes. While the proposed XML structure is feasible for most of the functional genomic data types, it can not be applied to high-throughput sequencing experiments. The usage of XML for the representation of such data might be counterproductive here, because XML is a human-readable format which adds lots of redundant text to the actual data. Therefore, in practice we do not transform such data sets to XML, but rather create metadata XML files for the search index that store processed data. The raw files (e.g. BAM files in the case of next generation sequencing or CEL files in the case of microarrays) are stored in the file system and are only referenced by the indexed metadata XML file.

One important issue within collaborative research groups is data security. Experimentalists need to be able to maintain in control of their raw data and study results need to be dealt with confidentially before they are published in a research journal. This can best be accomplished by securing the system with password protection and possibly also IP range restrictions at the web server configuration.

Also a more fine-grained user management can be realized by using the Foswiki user group functionality. Then, certain pages of the web site can be restricted to certain users or groups. Additionally, this concept could easily be extended to the central Solr index search so that particular search results would be restricted to specific users. For this purpose, the Solr-Search-Plugin would need to read the current user ID via the respective Foswiki variable and then filter the index results according to the logged in user. An overview of corresponding current and planned developments can be found at the DIPSBC homepage under the section 'Roadmap'.

Another advantage of the Foswiki collaboration platform worth mentioning is its intuitive data exchange function. At each page, users can upload files by clicking the 'Attach' button. Other users can then download the respective files. This has two important advantages compared to data sharing via e-mail: first, files that are too large for e-mail transmission can be shared; second, the reference file is stored only once at a central location, and if the file is changed, it can be downloaded again from the same location.

An important part of the proposed data integration system is the incorporation of data analysis results that add additional value to the raw experimental data and aid in the interpretation of these data. Currently, data analyses which lie beyond the capabilities of the Java applets need to be generated outside of the platform (see above use case *'Integration of experimental results from proteomic and transcriptomic data'*). However, for future development steps it might be worth considering the integration of an R interface that could enable the direct statistical processing of experimental data.

Our data integration system was already applied within several research projects, typically involving between 5 and 15 collaboration partners located at different sites. These small to medium sized projects likely represent the typical size for the majority of research projects. However, the system might as well be suited for larger collaborations, because the web server and Foswiki collaboration platform can still handle a lot more simultaneous accessions than would be generated by tens or even hundreds of participating users. This is proved by the fact that many companies use Foswiki as their intranet system, sometimes including thousands of web pages and high access rates.

As for scalability of the index machine, of course its search and index performance decreases with increasing numbers of stored documents. Nevertheless, the Solr/Lucene software library is optimized for very fast text queries on large amounts of data. E.g., the current index size of our data integration system amounts to almost 35 million indexed documents or 22.1 GB of physical storage, with Pubmed and UniProt records representing the major part. While indices of smaller size typically can be queried within split seconds, query times of this rather large index lie in the range of below one second for general queries and up to a few seconds for very complex queries. Therefore the system can be conveniently used to handle quite large amounts of documents. However, if larger index sizes are needed, as might be the case e.g. with meta-data of next-generation sequencing experiments, Solr/Lucene offers native support of distributed searches. For this purpose, a large index is split into several smaller indices on different machines, and thereby fast response times can be maintained.

All parts of the introduced system can be straightforwardly implemented. The basic system setup with the Foswiki user interface and the Solr backend can be achieved in less than one day by an experienced programmer. Also, an important advantage of the system is the fact that its components are open source. Therefore it can be modified and adjusted for specific functions.

Because of its flexibility, the system can easily incorporate additional or new data types like patient data, high-throughput sequencing data, or any other data types that will occur during future developments of experimental techniques. Adequate helper applications that make use of the underlying XML files can be developed or adapted efficiently in order to support the analysis of such new data. Therefore, the combination of a fast indexing machine with a web-based collaboration platform makes this system highly flexible, evolvable, scalable and easy to use for research collaborations.

## Conclusions

We developed DIPSBC, a systems biology data integration platform that utilizes a large number of XML-based exchange formats and connects primary data with higher-level data. The combination of a fast indexing machine with an online content management platform makes this system highly flexible and easy to use for research collaborations. Furthermore, the incorporation of helper applications is a powerful feature of the system, which distinguishes it from a mere data repository. Since all parts of the platform are open source, it can easily be modified and adjusted for specific functions.

### Availability and requirements

**Project name**: DIPSBC

**Project home page:**http://dipsbc.molgen.mpg.de

**Operating system(s)**: Platform independent

**Programming language(s)**: Perl, Java

**Other requirements**: Perl 5.8 or higher, Java 1.5 or higher, Foswiki web server, Solr/Lucene index

**License**: GNU GPL

**Any restrictions to use by non-academics**: None.

## Competing interests

None.

## Authors' contributions

FD and TK developed the system. CH, RY and AK adapted the system for specific collaboration projects. KS, HL and BML provided data sets for testing data integration and data collaboration. RH led the development and design of the system. FD and RH drafted the manuscript. All authors read and approved the final manuscript.

## Funding

This work was supported by the German Research Foundation (grants HE 4607/2-1 and HE 4607/3-1), the BMBF under its NGFN-Plus (01GS08111), NGFN-transfer (01GR0809) and MEDSYS programs (0315428A), and by the Max Planck Society.
